# Developmental changes of the impact of visual cues on ANS acuity across grades 1-5: Different patterns of visual cues on numerosity processing

**DOI:** 10.1177/20416695241259160

**Published:** 2024-06-05

**Authors:** Yike Tang, Ping Qian, Linlin Yan

**Affiliations:** 12646 Zhejiang Sci-Tech University, Hangzhou, China; 12646 Zhejiang Sci-Tech University, Hangzhou, China; 12646 Zhejiang Sci-Tech University, Hangzhou, China

**Keywords:** approximate number system, visual cues, non-symbolic numerosity comparison, school-aged children, facilitating effect, attenuated effect

## Abstract

Numerous studies have consistently demonstrated the presence of the approximate number system (ANS) throughout development. Research has also revealed that visual cues may influence the ANS acuity, which may change with age. However, most studies have drawn conclusions based on performance differences between incongruent and congruent trials, which may be confounded by an individual's ability to inhibit interference. Therefore, to examine the developmental changes of the impact of visual cues on ANS acuity, we utilized congruent trials with varying visual cues. Our sample comprised Chinese children from grade one to grade five. We manipulated the salience of numerical cues (numerical ratio) and visual cues (dot size) in a non-symbolic numerosity comparison task. The results revealed a discernible leap in development from first to third grade and first to fifth grade; however, this upward trajectory did not persist into the transition from third to fifth grade, where no appreciable advancement was observed. Moreover, we observed different effects of visual cues on the dot-comparison task depending on the numerical cues and age. Specifically, visual cues (i.e., dot size) only facilitated ANS acuity in older school-aged children when numerical cues were weakened. The results indicate the presence of two distinct magnitude representational systems—one for the numerical dimension and another for the non-numerical dimension—during development.

Humans and non-human animals have an intuitive sense of numerosity that allows them to estimate and compare quantities without language and counting. This capability relies on the approximate number system (ANS, Feigenson et al., 2004). The ANS serves as a foundational element for the development of our symbolic mathematical abilities and plays an important role in formal math learning (Halberda et al., 2012). Previous studies have shown that young children's non-symbolic numerical magnitude comparison was related to their symbolic mathematical ability (Fazio et al., 2014; Schneider et al., 2017). To assess the acuity of the ANS, researchers often use dot-numerosity comparison tasks, where participants are presented with two sets of dots simultaneously and asked to determine which set is more numerous. Performance indicators in these tasks include accuracy rate, correct response time (RT), and inverse efficient score (IES).^1^ These indicators provide valuable insights into individuals’ numerical processing capabilities and their potential impact on mathematical learning.

Indeed, the indicators used in the dot-numerosity comparison task may not solely measure ANS acuity. The dots in the task encompass numerical cues (e.g., numerical ratio) and visual cues (e.g., dot size). To mitigate the influence of visual cues, researchers often strive to control the visual characteristics of the dots while varying the numerical ratio of the dot arrays. Nonetheless, it is crucial to acknowledge that some residual impact of visual cues may persist. For example, controlling the size of a single dot could result in challenges in precisely controlling the overall area of the dot array when it changes only with the numerical ratio. Therefore, some researchers propose that quantity perception involves the integration of different sensory cues derived from dot stimuli, specifically numerical and visual cues. This theory is known as the sensory integration account (Gebuis & Reynvoet, 2012; Gebuis et al., 2016; Leibovich & Henik, 2014). In a study by Leibovich and Henik (2014), participants’ performances were compared in tasks involving numerosity comparison in dot arrays and area comparison in squares. The researchers found that performance in the numerosity comparison task conformed to Weber's law (i.e., a historically important psychological principle quantifying the perception of changes in a given stimulus), while performance in the area comparison task violated it.

However, some researchers propose a competing process account (Clayton & Gilmore, 2014; Clayton et al., 2019). They suggest that there is a competitive relationship between processing numerical cues and other non-numerical visual cues. Dot comparison tasks typically include both congruent and incongruent trials. During congruent trials, visual cues such as the dot size of the array are positively correlated with numerosity. Conversely, incongruent trials feature a negative correlation between dot size and numerosity, where the array with fewer dots consists of larger dots. Individuals must inhibit numerically irrelevant and misleading visual cues. From this perspective, it appears likely that the congruency effect in dot comparison tasks may partially assess inhibitory control skills. According to Wilkey et al. (2017), brain regions (i.e., left anterior cingulate, left precentral gyrus, left intraparietal sulcus, and right superior parietal lobe) previously implicated in processing numerical cues did not exhibit variation in response to the congruency effect. However, they observed selective recruitment of the right inferior frontal gyrus (IFG) during incongruent trials. The IFG has been shown to correlate with inhibitory control ability, indicating its role in suppressing automated behaviors in incongruent trials (Hampshire et al., 2010). Thus, the question of whether quantity perception relies solely on numerical cues or a combination of numerical cues and visual cues remains unresolved.

Moreover, the inconsistent findings regarding the influence of visual cues on quantity perception may be influenced by age. Previous studies have indicated that individuals’ dependence on visual cues varies across different developmental stages (Clayton et al., 2019). For example, studies have shown that preschool-aged children rely heavily on visual cues to perceive quantity, while older children and adults rely more on numerical information (Rousselle & Noël, 2008; Viarouge et al., 2019). Rousselle and Noël (2008) found that in the congruent conditions (when the array had more dots and a larger filled area), visual cues enhanced the perception of quantity in 6-year-old children. However, in incongruent conditions (when the array had more dots but a smaller filled area), visual cues interfered with their perception of quantity. This suggests that preschoolers rely on visual cues to perceive quantity. Wilkey et al. (2022) investigated the performance of 7-year-olds in the dot-numerosity comparison task twice with a 5-month interval. They found that the accuracy rate increased in both incongruent and congruent conditions. This indicates that as children grow older, their ability to discriminate non-symbolic numbers improves due to a more sophisticated magnitude representation. Furthermore, Viarouge et al. (2019) used the negative priming paradigm and found that in incongruent conditions (i.e., when the array had more dots and less surface area), discrimination accuracy was significantly lower in children aged 7–8 compared to adults. This suggests that as individuals get older, the interference of visual cues on quantity perception decreases gradually. Currently, few researchers have directly investigated the change in reliance on visual cues for quantity perception in children aged 6–12 during dot-numerosity comparison tasks.

However, previous studies often focused on the performance differences between congruent and incongruent trials to explore the impact of visual cues on the ANS acuity of children, introducing the confounding variable of inhibitory control. As previously noted, Wilkey et al. (2017) discovered that the differential brain activity between congruent and incongruent trials was localized to the IFG, a region associated with inhibition. Additionally, Skagenholt et al. (2021) reported that the primary difference in brain activation between children and adults during non-symbolic and symbolic comparison tasks was observed in regions commonly linked to domain-general cognitive control. Consequently, merely comparing the differences between congruent and incongruent trials does not suffice to elucidate how visual cues affect behavior in the dot-comparison task or how this influence evolves with age. Moreover, some researchers have identified an accumulative effect of visual cues on dot comparison (Bonn & Odic, 2024; Leibovich & Ansari, 2017). Leibovich et al. found brain region did not modulate according to task difficulty but rather in response to the accumulation of non-numerical magnitudes. To gain a clearer understanding of the role of visual cues in the dot-comparison task, our study utilizes congruent trials with varying levels of salience. By exclusively examining congruent trials, this study aims to eliminate the confounding factor of inhibition and offer deeper insights into the role of visual cues in quantity perception during childhood.

Given that the reliance on visual cues for the dot-numerosity comparison varies with age, our experiment aimed to explore how visual cues affect ANS acuity during elementary school, and how this influence develops with increasing grades. We recruited children from grades one, three, and five to complete a dot-numerosity comparison task. In this task, we manipulated the salience (weak vs. strong) of numerical and visual cues of the dots. Numerical cues were manipulated by varying the ratio of the number of dots in two arrays. Visual cues were manipulated by altering the average size of the dots in two arrays. In the weak-visual-cues condition, the average size was the same in both arrays. In the strong-visual-cues condition, the average size was larger in the array with more dots. Additionally, in both visual-cues conditions, the total area was larger in an array with more dots.

If the sensory integration account holds true, optimal performance would occur when strong numerical cues are combined with strong visual cues (i.e., strong numerical + strong visual), while the poorest performance would result from combining weak numerical cues with weak visual cues (i.e., weak numerical + weak visual). Performance under other conditions (i.e., strong numerical + weak visual, or weak numerical + strong visual) would fall between these two extremes. Based on previous developmental studies (Kuzmina et al., 2020; Leibovich & Ansari, 2017; Park et al., 2018), we predicted that visual cues would equally facilitate children's quantity processing as they grow older. On the other hand, if the competing processes account is valid, there would be no difference in performance between combining strong numerical cues with strong visual cues (i.e., strong numerical + strong visual) and using a single strong cue (i.e., strong numerical + weak visual, or weak numerical + strong numerical). Based on previous developmental studies (Clayton et al., 2019; Defever et al., 2013; Odic, 2018), we predicted that the influence of visual cues on children's quantity processing would vary across the three grades.

## Methods

### Participants

Sixty-six students (33 males) were recruited as participants from an elementary school in Hangzhou, including 22 participants from Grade One (*M*_age _= 7.45 years, *SD*_age _= 0.67), 22 participants from Grade Three (*M*_age _= 9.32, *SD*_age _= 0.78) and 22 participants from Grade Five (*M*_age _= 11.23, *SD*_age _= 0.75). This study was approved by the Human Research Ethics Committee of Zhejiang Sci-tech University. Participants gave informed consent and were rewarded for participation.

To justify the number of participants for the current study, we performed a Power analysis by MorePower 6.0 (Campbell & Thompson, 2012) to estimate sample size with the partial *η*² and design factors from previously published studies (Piazza et al., 2018; Pina et al., 2015; Viarouge et al., 2019). Other parameters of Power Analysis were set as follows: a significance level of 0.05, a power value of 0.8, and the test as an “ANOVA test.” The results indicated that the estimated sample size ranges from 10 to 26 for each group (*Mean *= 20/group). Therefore, 22 participants for each group in the current study were sufficient to examine the role of visual cues in numerosity estimation acuity across school age.

### Stimuli

**Figure 1.**
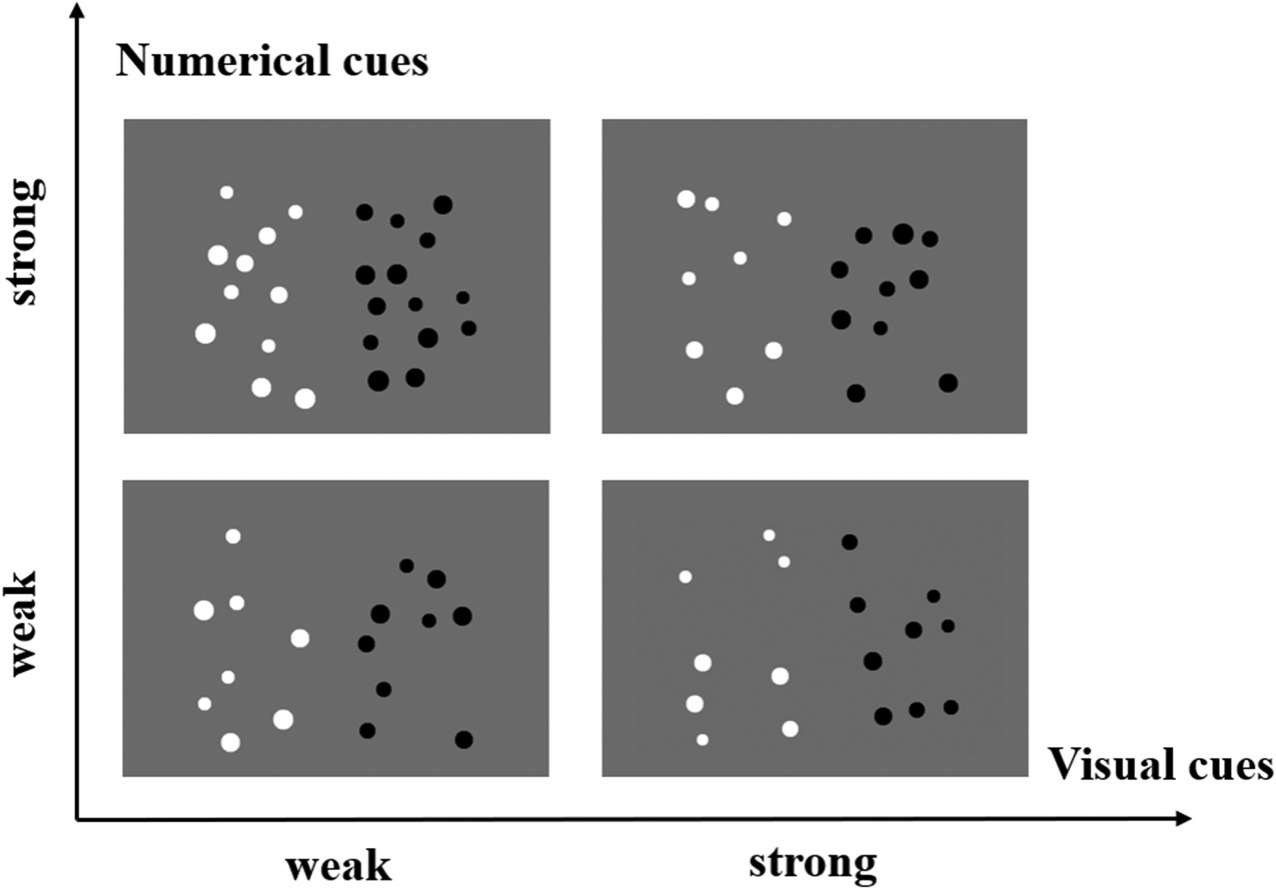
Examples of dot arrays administered in the Panamath task.

*Note:* The visual cues were congruent with the numerical cues. The only distinction lies in the levels of congruency between the visual cues and numerical cues. The dot arrays were generated with variations of the visual cues (weak: the total surface area was proportional to the number of dots, average dot size equated; strong: total surface area and average dot size were proportional to the number of dots) and numerical cues^2^ (weak: the numerical ratio ranged from 1.1 to 1.2; strong: the numerical ratio ranged from 1.2 to 1.3; the numerical ratio was based on the ratio of black dots and white dots as examples).

The stimuli created by the Panamath software (version 1.22, Halberda et al., 2008) were pairs of dot arrays. The pairs of dot arrays were presented on the 14-inch laptop screen with a resolution of 1366 × 768 pixels and a refresh rate of 60 Hz. Each array of dots was presented simultaneously side-by-side on a grey background (white dots on the left, black dots on the right, see Figure 1) at a visual angle of 5.7° from a central fixation cross. The absolute number of dots ranged from 5 to 21.

The dots were generated with two within-subject factors: numerical cues with two levels (weak vs. strong) and visual cues with two levels (weak vs. strong). For the weak-numerical-cues items, the ratio of the dots-array pairs presented ranged from 1.1 to 1.2. For the strong-numerical-cues items, the ratio of the dots-array pairs presented ranged from 1.2 to 1.3. For the weak-visual-cues items, the total surface area of the dots was proportional to the number of dots in each array, but the average size of the dots in the two arrays was equated. For the strong-visual-cues items, both the total surface area and the average size of the dots were proportional to the number of dots in the array.

### Procedures

Participants were asked to complete a dot-numerosity comparison task. They had to determine which side had more dots based on a brief display of black dots and white dots on the screen. Each trial began with a fixation point presented at the center of the screen until participants pressed the space bar. Then a pair of dot arrays was presented simultaneously side-by-side on a grey background for 600 ms. Participants were prompted to press “F” for more white dots or “J” for more black dots, then press the space bar to the next trial.

Participants completed a total of 60 trials in the dot-numerosity comparison task—2 (Numerical Cues: weak vs. strong) × 2 (Visual Cues: weak vs. strong). Another 60 trials with ratios ranging from 1.3 to 2.0 are generally deemed relatively easy for children aged 7 and above (Halberda & Feigenson, 2008). As such, these trials will be used as fillers and not included in the analysis. To ensure consistency, we used the same ratio distribution for the three grades. The total duration of the task was approximately 10 min. The experiment started with four practice trials.

### Calculation of Inverse Efficiency Score

Firstly, we will examine how numerical cues and visual cues influence the comparison of quantity between two arrays using accuracy and reaction time (RT) as measures. In the numeracy literature, it is common to use either accuracy or RT individually, but rarely are both employed simultaneously. However, since individuals may prioritize either accuracy or speed (Ratcliff et al., 2015), it is essential to utilize both variables concurrently to interpret data on numeracy processes and representations.

Taking into account the speed-accuracy trade-off in cognitive processing, Townsend and Ashby (1983) proposed the IES as a composite index that combines RT and accuracy rate. The IES is calculated as the ratio of correct RT to accuracy rate (IES = correct RT/Accuracy Rate). This integrated measure provides a more comprehensive assessment and enables a more reliable conclusion, particularly when there is a high and linear correlation between RT and error rate (Statsenko et al., 2020).

## Results

### Accuracy Rate

To assess ANS acuity, the accuracy rate (proportion of correct answers) was calculated for each condition and each participant. The descriptive statistics of the accuracy rate in the dot-numerosity comparison task are presented in Table 1.

**Table 1.** Descriptive Statistics for Accuracy Rate (%, M ± SE) in the Dot-Numerosity Comparison Task.

*Grade*	*All* *(M ± SE)*	*Weak* *Numerical cues*		*Strong* *Numerical cues*	
		*Weak visual cues*	*Strong Visual cues*	*Weak visual cues*	*Strong Visual cues*
One	69.55 ± 1.44	60.91 ± 3.17	67.27 ± 1.91	74.55 ± 2.80	75.45 ± 2.95
Three	73.79 ± 1.44	68.79 ± 2.46	70.91 ± 2.69	80.00 ± 2.19	75.45 ± 2.53
Five	76.97 ± 1.44	67.27 ± 2.32	78.48 ± 1.91	81.51 ± 2.52	80.61 ± 2.14

We performed a repeated-measures ANOVA on the accuracy rate with the factors of numerical cues (weak and strong), visual cues (weak and strong), and grades (one, three, and five). There was a main effect of grades, *F* (2,63) = 6.73, *p *= .002, *η*_p_^² ^= 0.18, a main effect of numerical cues, *F* (1,63) = 37.20, *p *< .001, *η*_p_^² ^= 0.37, and a marginal main effect of visual cues, *F* (1,63) = 3.76, *p *= .057, *η*_p_^² ^= 0.056. Moreover, there was an interaction between numerical cues and visual cues, *F* (1,63) = 9.69, *p *= .003, *η*_p_^² ^= 0.13. There were no other significant effects [numerical cues × grades: *F* (2,63) = 0.43, *p *= .65, *η*_p_^² ^= 0.013; visual cues × grades: *F* (2,63) = 2.17, *p *= .12, *η*_p_^² ^= 0.065; visual cues × numerical cues × grades: *F* (2,63) = 0.62, *p *= .54, *η*_p_^² ^= 0.019].

The main effect of grades was analyzed using the Bonferroni post-hoc test. The results showed a significant difference in accuracy rate between grade one (*M ± SE_grade-one _*= 69.55% ± 1.44%) and grade five (*M ± SE_grade-five _*= 76.97% ± 1.44%), with a *p*-value of .002. However, no significant difference was found between grades one and three (*M ± SE_grade-three _*= 73.79% ± 1.44%, *p *= .12), or between grades three and five (*p *= .37). These findings suggest a grade-related improvement in ANS acuity between grades one and five.

**Figure 2.**
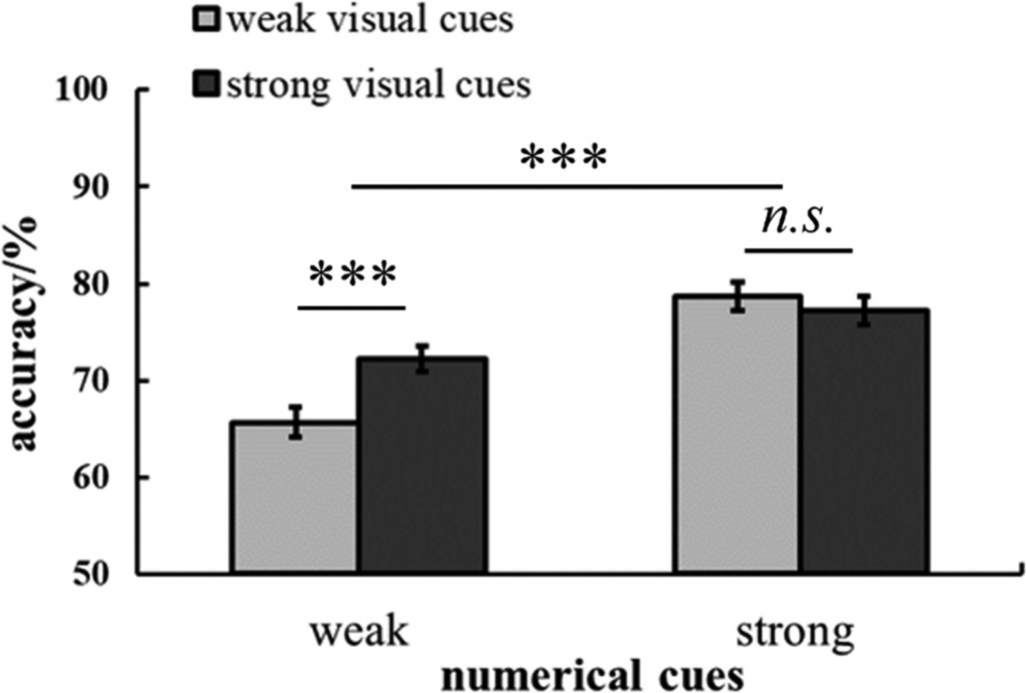
The accuracy rate in dot array comparison by visual cues and numerical cues.

*Note:* Black bars represent strong visual cues while grey bars represent weak ones. Error bars indicate *SE*s and *** indicate *p *< .001.

For the interaction effect between numerical cues and visual cues, the simple effect analysis revealed an effect in the weak numerical cues, *F* (1,65) = 13.38, *p *< .001, *η*_p_^² ^= 0.17. The accuracy in strong visual cues (*M ± SE_strong-visual-cues _*= 72.22% ± 1.38%) was higher than that in weak visual cues (*M ± SE_weak-visual-cues _*= 65.66% ± 1.58%). But there was no difference in accuracy between the weak visual cues (*M ± SE_weak-visual-cues _*= 78.69%± 1.48%) and strong visual cues (*M ± SE_strong-visual-cues _*= 77.17% ± 1.49%) when the numerical cues were strong, *F* (1,65) = 0.63, *p *= .43, *η*_p_^² ^= 0.01. On the other hand, the accuracy in strong numerical cues (*M ± SE_strong-numerical-cues _*= 78.69% ± 1.48%) was higher than that in weak numerical cues (*M ± SE_weak-numerical-cues _*= 65.66% ± 1.55%) when the dots array with weak visual cues, *F* (1,65) = 39.94, *p *< .001, *η*_p_^² ^= 0.38. And the accuracy in strong numerical cues (*M ± SE_strong-numerical-cues _*= 77.17% ± 1.49%) was higher than that in weak numerical cues (*M ± SE_weak-numerical-cues _*= 72.22% ± 1.38%) when the dots array with strong visual cues, *F* (1,65) = 39.94, *p *< .001, *η*_p_^² ^= 0.38. The results indicate that strong numerical cues improve the ANS acuity regardless of the presence of visual cues. However, strong visual cues only assist children in comparing the numerosity of dots when the dot arrays have weak numerical cues. These results are shown in Figure 2.

To examine how the impact of visual cues differs across different grades, we used the visual effect (visual effect_ACC = ACC_strong-visual-cues_ − ACC_weak-visual-cues_) as an index that represented the impact of visual cues on ANS acuity. The visual effect_ACC greater than zero indicated the facilitating effect of visual cues and the value less than zero indicated the attenuated effect of visual cues. The value close to zero indicated no visual effect on ANS acuity.

**Figure 3.**
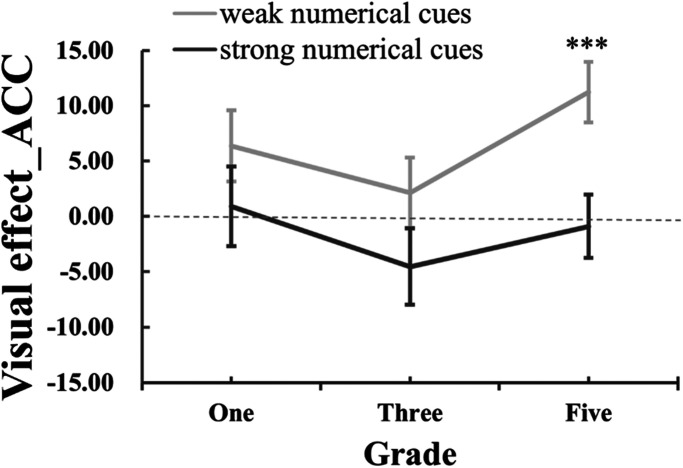
The visual effect on accuracy rate under different numerical cues across three grades.

*Note:* The visual effect (visual effect_ACC = ACC*_strong-visual-cues_* − ACC*_weak-visual-cues_*) is an index that represented the impact of visual cues on ANS acuity. The values of the visual effect represent the magnitude of visual cues effects. Positive values represent facilitating effect of visual cues while negative values represent the attenuated effect of visual cues. The black line represents strong numerical cues while the grey line represents weak numerical cues. Error bars indicate *SE*s and *** indicate vs. zero, *p *< .001.

For the visual effect_ACC (see Figure 3), we initially conducted a repeated-measures ANOVA. The results demonstrated a significant main effect of numerical cues, *F* (1,63) = 9.69, *p *= .003, *η*_p_^² ^= 0.13. Specifically, the visual effect was smaller for trials with strong numerical cues (*M ± SE_strong-numerical-cues _*= −1.52 ± 1.91) compared to trials with weak numerical cues (*M ± SE_weak-numerical-cues _*= 6.57 ± 1.76). There were no other significant effects [numerical cues × grades: *F* (2,63) = 0.62, *p *= 0.54, *η*_p_^² ^= 0.019; grades: *F* (2,63) = 2.17, *p *= 0.12, *η*_p_^² ^= 0.065]. The ANOVA result on the visual effect showed that visual cues have significantly more impact on trials with weak numerical cues.

To investigate the influence of visual cues on quantity comparison, we conducted one-sample *t*-tests to determine if the visual effect_ACC significantly differed from zero for the two types of numerical cues. The results revealed that the visual effect for dot arrays with weak numerical cues was significantly greater than zero [*t*(65) = 3.66, *p *= .001], indicating that visual cues had an impact when weak numerical cues were present. However, the visual effect for dot arrays with strong numerical cues did not deviate significantly from zero [*t*(65) = −0.8, *p *= .43], suggesting that visual cues had little influence when strong numerical cues were present. Further analysis specifically focused on the visual effect of fifth-grade children. We found that for these children, the visual effect was significantly greater than zero when comparing the dots with weak numerical cues [*t*(21) = 4.07, *p *= .001]. However, no other visual effect significantly deviated from zero [grade-one_weak-numerical_: *t*(21) = 1.98, *p *= .06; grade-one_strong-numerical_: *t*(21) = 0.25, *p *= .80; grade-three_weak-numerical_: *t*(21) = 0.67, *p *= .51; grade-three_strong-numerical_: *t*(21) = −1.32, *p *= .20; grade-five_strong-numerical_: *t*(21) = −0.32, *p *= .75]. These findings indicate that visual cues facilitated the judgment of fifth-grade children, particularly when the numerical cues were weak. This underscores the tendency of older children to rely more on visual information when numerical cues are less informative.

In sum, our findings indicate that ANS accuracy improves with the going up of the grades, suggesting that ANS acuity is influenced by grade-related improvement. We also found that visual cues only facilitate ANS accuracy in trials with weak numerical cues, especially among children in grade five. This suggests that visual cues (i.e., dots with larger areas) can improve ANS acuity when numerical cues are less reliable.

### Correct RT

To assess ANS efficiency, the correct RT (RT: mean RT of the correct responses) was calculated for each condition and each participant. The descriptive statistics of the correct RT are presented in Table 2.

**Table 2.** Descriptive Statistics for Correct RT (ms, M ± SE) in the Dot-Numerosity Comparison Task.

*Grade*	*ALL* *(M ± SE)*	*Weak* *Numerical cues*		*Strong* *Numerical cues*	
		*Weak visual cues*	*Strong Visual cues*	*Weak visual cues*	*Strong Visual cues*
One	1507 ± 75	1450 ± 83	1648 ± 136	1468 ± 81	1462 ± 99
Three	1142 ± 75	1203 ± 84	1086 ± 68	1181 ± 81	1096 ± 98
Five	985 ± 75	995 ± 61	946 ± 43	990 ± 51	1007 ± 69

We performed a repeated-measures ANOVA on the correct RT with the factors of numerical cues (weak and strong), visual cues (weak and strong), and grades (one, three, and five). There was a main effect of grades, *F* (2,63) = 12.66, *p *< .001, *η*_p_^² ^= 0.29, an interaction effect between visual cues and grades, *F* (2,63) = 4.28, *p *= .018, *η*_p_² = 0.12 and a three-way interaction effect, *F* (2,63) = 4.18, *p *= .02, *η*_p_² = 0.12. There were no other effects [numerical cues: *F* (1,63) = 1.02, *p *= .32, *η*_p_² = 0.016; visual cues: *F* (1,63) = 0.066, *p *= .8, *η*_p_² = 0.001; numerical cues × visual cues, *F* (2,63) = 0.72, *p *= .4, *η*_p_² = 0.011; numerical cues × grades: *F* (2,63) = 2.65, *p *= .079, *η*_p_² = 0.078].

The main effect of grades was analyzed using the Bonferroni post-hoc test. The results showed a significant difference in RT between grade one (*M *± *SE _grade-one _*= 1507 ms ± 75 ms) and grade three (*M *± *SE _grade-three _*= 1142 ms ± 75 ms) with a *p*-value of .003. There was also a significant difference in RT between grade one and grade five (*M *± *SE _grade-five _*= 985 ms ± 75 ms) with a *p-value* of .001. However, there was no significant difference in RT between grade three and grade five (*p *= .43). These findings indicate that there is a grade-related improvement in ANS efficiency, with a significant reduction in latency observed between grade one and grade three, as well as between grade one and grade five.

**Figure 4.**
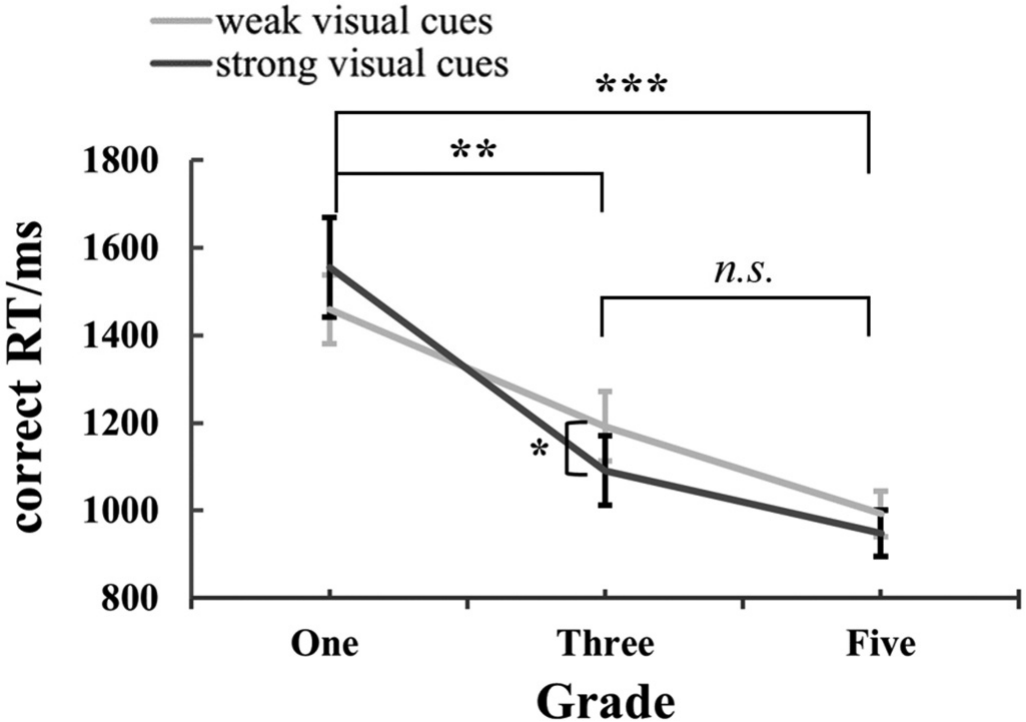
The correct RT in dot array comparison by visual cues and grade.

*Note:* Black line represents strong visual cues while the grey line represents weak ones. Error bars indicate *SE*s and * indicates *p *< .05.

For the interaction effect between visual cues and grades, the simple effect analysis revealed that only the correct RT of grade three children showed a significant difference between weak and strong visual cues (grade three: *M ± SE_weak-visual-cues _*= 1192 ± 80, *M ± SE_strong-visual-cues _*= 1091 ± 80, *p *= .02). But there was no significant difference between weak and strong visual cues for other grades (grade one: *M ± SE_weak-visual-cues _*= 1459 ± 78, *M ± SE_strong-visual-cues _*= 1555 ± 114, *p *= .15; grade five: *M ± SE_weak-visual-cues _*= 992 ± 52, *M ± SE_strong-visual-cues _*= 977 ± 53, *p *= .65). On the other hand, irrespective of weak visual cues (grade one vs. grade three: *p *= .005; grade one vs. grade five: *p < *.001; grade three vs. grade-five, *p *= .15) or strong visual cues (grade-one vs. grade-three: *p *= .002; grade-one vs. grade-five: *p < *.001; grade-three vs. grade-five = .77), the correct RT from grade one was the longest, with no significant difference between grade three and grade five. The results are shown in Figure 4.

To better understand how the impact of visual cues changes across grades, we used the visual effect (visual effect_RT = RT_strong-visual-cues_ − RT_weak-visual-cues_) as an index that represented the impact of visual cues on ANS efficiency. In contrast with the visual effect_ACC, the visual effect_RT less than zero indicated the facilitating effect of visual cues and the value greater than zero indicated the attenuated effect of visual cues. The value close to zero indicated no visual effect on ANS efficiency.

**Figure 5.**
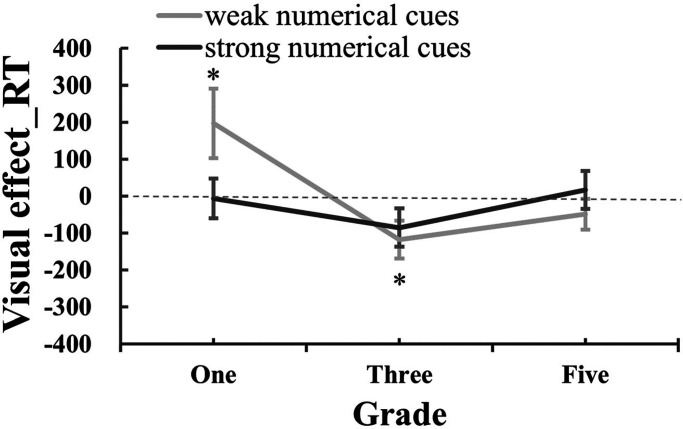
The visual effect on correct RT under different numerical cues across three grades.

*Note:* The visual effect (visual effect_RT = RT*_strong-visual-cues_* − RT*_weak-visual-cues_*) is an index that represented the impact of visual cues on ANS efficiency. The values of the visual effect represent the magnitude of visual cues effects. Positive values represent the attenuated effect of visual cues while negative values represent the facilitating effect of visual cues. The black line represents strong numerical cues while the grey line represents weak numerical cues. Error bars indicate *SE*s and * indicates vs. zero, *p *< .05.

For the visual effect_RT (see Figure 5), we initially conducted a repeated-measures ANOVA, which did not yield any significant effects [numerical cues: *F* (1,63) = 2.09, *p *= .15, *η*_p_^² ^= 0.032; numerical cues × grades: *F* (2,63) = 2.30, *p *= .11, *η*_p_^² ^= 0.068; grades: *F* (2,63) = 0.62, *p *= .54, *η*_p_^² ^= 0.019]. Subsequently, one-sample *t*-tests were conducted to examine whether the visual effect_RT significantly deviated from zero for each condition. The results revealed that the visual effect in RT for grade-one children in weak numerical trials was significantly higher than zero [*t*(21) = 2.11, *p *= .047), while it was significantly lower than zero for grade-three children in weak numerical trials [*t*(21) = −2.29, *p *= .032). No other visual effect values in RT showed significant deviations from zero [grade_one_strong-numerical_: *t*(21) = −0.72, *p *= .48; grade-three_strong-numerical_: *t*(21) = −1.33, *p *= .20; grade-five_weak-numerical_: *t*(21) = 0.23, *p *= .82; grade-five_strong-numerical_: *t*(21) = −0.77, *p *= .45]. The findings suggest that visual cues only influence the correct RT in weak numerical trials. Specifically, grade-one children displayed an attenuated effect of visual cues, whereas grade-three children exhibited the facilitating effect of visual cues.

In sum, our study revealed that the correct RT shortened with the going up of the grades, which suggested that ANS efficiency was a grade-related improvement. Additionally, we found that visual cues only influenced the ANS efficiency in weak-numerical cues trials, especially for the children in grade one and grade three. Specifically, grade one children showed the attenuated effect of visual cues, and grade three children showed the facilitating effect of visual cues. The results indicate that visual cues (i.e., dots with larger areas) had a slowing down on the ANS efficiency of grade one children, but facilitated the ANS efficiency of grade three when the numerical cues were weak.

### Inverse Efficiency Score

Traditionally, accuracy and RT have been considered as separate metrics to assess performance in cognitive tasks. However, this approach overlooks the potential trade-off between accuracy and RT, as well as variability stemming from individual ability and task difficulty (Dodonova & Dodonov, 2013). To address this issue and gain a more comprehensive understanding of participants’ performance, the IES (IES = correct RT/Accuracy Rate) was calculated to indicate the ANS acuity (see Table 3).

**Table 3.** Descriptive Statistics for Inverse Efficiency Score (IES, M ± SE) in the Dot-Numerosity Comparison Task.

*Grade*	*ALL* *(M ± SE)*	*Weak* *Numerical cues*		*Strong* *Numerical cues*	
		*Weak visual cues*	*Strong Visual cues*	*Weak visual cues*	*Strong Visual cues*
One	2238 ± 105	2466 ± 157	2456 ± 188	2014 ± 129	2016 ± 185
Three	1573 ± 105	1203 ± 84	1086 ± 68	1181 ± 81	1096 ± 98
Five	1310 ± 105	995 ± 61	946 ± 43	990 ± 51	1007 ± 69

We performed a repeated-measures ANOVA on the IES with the factors of numerical cues (weak and strong), visual cues (weak and strong), and grades (one, three, and five). There was a main effect of grades, *F* (2,63) = 20.63, *p *< .001, *η*_p_^² ^= 0.4, and a main effect of numerical cues, *F* (1,63) = 27.62, *p *< .001, *η*_p_^² ^= 0.31. Moreover, there was an interaction between numerical cues and grades, *F* (1,63) = 4.63, *p *= .013, *η*_p_^² ^= 0.13. There were no other significant effects [visual cues: *F* (1,63) = 2.1, *p *= .15, *η*_p_^² ^= 0.032; visual cues × grades: *F* (2,63) = 0.57, *p *= .57, *η*_p_^² ^= 0.018; visual cues × numerical cues: *F* (2,63) = 3.24, *p *= .077, *η*_p_^² ^= 0.049; visual cues × numerical cues × grades: *F* (2,63) = 0.89, *p *= .42, *η*_p_^² ^= 0.028].

The main effect of grades was analyzed using the Bonferroni post-hoc test. The results indicated that grade one had a significantly larger IES (*M *± *SE _grade-one _*= 2238 ± 105, *p*s* *< .001) compared to both grade three (*M *± *SE _grade-three _*= 1573 ± 105) and grade five (*M *± *SE _grade-five _*= 1310 ± 105). However, there was no significant difference in IES between grade three and grade five (*p *= .25). These findings suggest that there is the lowest level of ANS acuity in grade one, but no difference was observed between grade three and grade five.

For the main effect of the numerical cues, the children showed a higher IES when presented with weak numerical cues than with the strong numerical cues (*M *± *SE _weak-numerical-cues _*= 1830 ± 65, *M *± *SE _strong-numerical-cues _*= 1584 ± 66). This indicates that the children performed better when the numerical cues were stronger.

**Figure 6.**
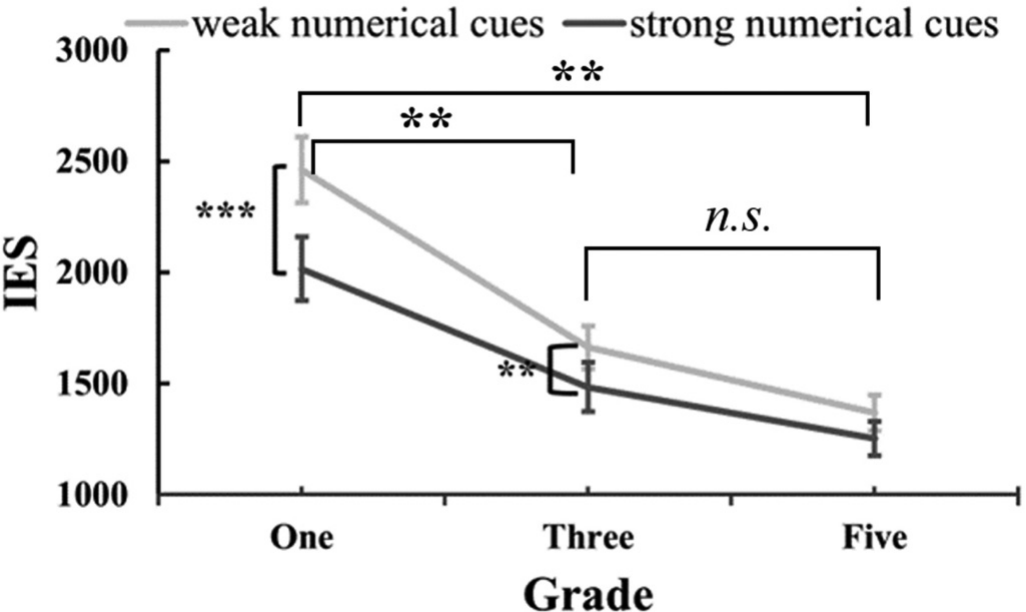
The IES in dot array comparison by numerical cues and grades.

*Note:* Black line represents strong visual cues while the grey line represents weak ones. Error bars indicate *SE*s, ** indicates *p *< .01, and *** indicates *p *< .001.

For the interaction between numerical cues and grades (Figure 6), the simple effect analysis showed that grade one and grade three children showed higher IES in weak numerical cues than in strong numerical cues (grade one: *p *= .001; grade three: *p *= .007). Grade five children showed no significant difference between weak and strong numerical (*p *= .079). On the other side, grade one showed higher IES than those of grade three and grade five children (grade one vs. grade three: *p *< .001; grade one vs. grade five: *p *< .001), but no significant difference in IES between grade three and grade five children (*p *= .29) regardless of the numerical cues.

**Figure 7.**
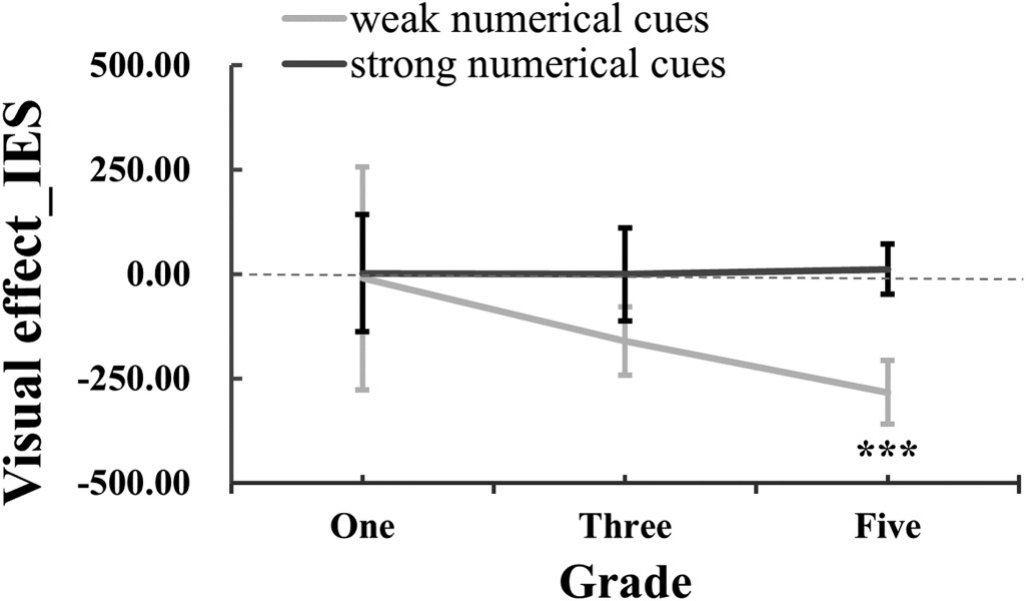
The visual effect on IES under different numerical cues across three grades.

*Note:* The visual effect (visual effect_IES = IES*_strong-visual-cues_* − IES*_weak-visual-cues_*) is an index that represented the impact of visual cues on ANS acuity. The values of the visual effect represent the magnitude of visual cues effects. Positive values represent the attenuated effect of visual cues while negative values represent the facilitating effect of visual cues. The black line represents strong numerical cues while the grey line represents weak numerical cues. Error bars indicate *SE*s and *** indicates vs. zero, *p *< .001.

For the visual effect_IES (see Figure 7), we initially performed a repeated-measures ANOVA. The results showed no significant effects [numerical cues: *F* (1,63) = 0.043, *p *= .84, *η*_p_^² ^= 0.001; numerical cues × grades: *F* (2,63) = 0.41, *p *= .67, *η*_p_^² ^= 0.013; grades: *F* (2,63) = 0.02, *p *= .98, *η*_p_^² ^= 0.001]. To further investigate the impact of visual cues on judgments, one-sample *t*-tests were used to examine whether the visual effect_IES differed from zero for each condition. Interestingly, the visual effect of grade-five children in weak numerical trials was found to be significantly lower than zero [*t*(21) = −3.74, *p *= .001). This suggests that grade five children experienced a facilitating effect from the visual cues in weak numerical trials. Conversely, no other visual effect values deviated significantly from zero [grade-one_weak-numerical_: *t*(21) = −0.077, *p *= .94; grade-one_strong-numerical_: *t*(21) = −0.43, *p *= .67; grade-three_weak-numerical_: *t*(21) = −1.01, *p *= .32; grade-three_strong-numerical_: *t*(21) = 0.20, *p *= .84; grade-five_strong-numerical_: *t*(21) = −0.74, *p *= .47 ]. These findings highlight that grade five children specifically benefited from the presence of visual cues in weak numerical trials.

In sum, our findings indicated a decrease in IES as grades increased, particularly from grade one to grade three and grade five. This suggests that ANS acuity improves as grades go up, but the rate of improvement slows down with higher grades. Additionally, children demonstrated better performance when strong numerical cues were present, regardless of the presence of visual cues. Furthermore, it was observed that visual cues only facilitated the IES of grade five when the numerical cues were weak. This suggests that visual cues (i.e., dots with larger areas) can enhance the ANS acuity in older children, especially when the numerical cues are less prominent.

## Discussion

This study aimed to investigate the influence of visual cues on ANS acuity and developmental changes from grade one to grade five. Firstly, we observed an interaction between visual cues and numerical cues in quantity processing. Specifically, we found that visual cues had a positive impact on quantity comparison only when the numerical cues were not salient. However, when numerical cues were salient, we did not observe any notable influence of visual cues on quantity comparison. Secondly, ANS acuity improved from grade one to grade five, with the effects of visual cues on quantity processing showing variation across the three grades. Specifically, grade five children demonstrated a facilitating effect of visual cues on accuracy rate and IES. Grade three children exhibited a facilitating effect of visual cues in correct RT, while grade one children showed the attenuated effect of visual cues in correct RT.

Firstly, we found an interaction between numerical and visual cues in quantity processing. Specifically, we observed that visual cues facilitate the ANS acuity in school-aged children when numerical cues are weak. However, in cases where numerical cues are strong, we did not observe an obvious effect of visual cues on the ANS acuity of these children. The results align with the competing processes account (Clayton et al., 2019; Rodriguez & Ferreira, 2023), which suggests a competitive relationship between numerical processes and other processes driven by non-numerical visual cues. According to this theory, strong visual cues facilitate quantity comparison when numerical cues are not salient, while strong numerical cues enhance quantity comparison irrespective of the salience of visual cues. Leibovich et al. (2016) used a dot comparison task to test whether different brain regions were responsible for processing numerical and non-numerical dimensions of the dots. They found asymmetric processing of numerical and non-numerical magnitudes in the brain. Overall, these findings indicate the presence of two distinct representation systems for the numerical dimension and the non-numerical dimension.

Secondly, we observed an improvement in ANS acuity from grade one to grade five. Moreover, the impact of visual cues on quantity processing varied across different grades. The findings are consistent with previous evidence from Defever et al. (2013). They found that grade one students did not show a visual effect for both weak and strong numerical ratios, while grade three and grade six students exhibited a visual effect for weak ratios and no visual effect for strong ratios. This may be attributed to two explanations. The first explanation is that the task of comparing dots with weak numerical cues is more challenging for younger children, making it harder for them to allocate attention to both numerical and non-numerical visual cues simultaneously. The attention systems of young children are not fully developed to divide their focus across multiple dimensions, but this capability improves with age (Courage et al., 2015). Thus, grade five children demonstrated a facilitating effect of visual cues on quantity processing (evidenced by ACC and IES), while grade one children showed an attenuated effect of visual cues on quantity processing (evidenced by RT). The second explanation is related to asymmetric developmental rates of numerical and non-numerical magnitude representations. Odic (2018) showed that the disparity in discrimination abilities between numerosity and area increases and peaks around the age of 7, then decreases and stabilizes around the age of 11. This suggests that the development of numerical and non-numerical representation systems follows different trajectories. Although these explanations involve attention system maturation and developmental trajectories, the results further support the notion of separate systems for numerical and non-numerical representations from a developmental perspective.

The two lines of evidence presented above challenged the sensory integration account. According to this theory, optimal performance would be expected when strong numerical cues are combined with strong visual cues. Additionally, it was predicted that the impact of visual effects on quantity processing would increase as grades increased. However, our findings did not align with these predictions. Instead, we only observed optimal performance when the numerical cues are salient, suggesting that numerical cues are encoded with greater prominence compared to visual cues. Moreover, we found that the influence of visual effects on quantity processing varied across grades. Specifically, in the absence of salient numerical cues, grade one children showed an attenuating effect of visual cues on numerosity comparison (evidenced by RT), while grade five children exhibited an improvement in overall performance of numerosity comparison with visual cues. Hendryckx et al. (2021) discovered that adults flexibly utilize visual cues to enhance quantity processing, indicating that children gradually start employing visual cues to assist in quantity processing.

There are two limitations to this study. First, we solely focused on manipulating dot size and did not consider other non-numerical visual cues, particularly the convex hull. Prior research has shown that the convex hull affects quantity processing in children aged 5 to 14 years to a similar extent (Gilmore et al., 2016). The convex hull and other visual cues, which were not controlled in our research, could also introduce bias to the results. The second limitation is that the improvements in number acuity and area acuity during development may partially reflect enhancements from math education (Nys et al., 2013; Piazza et al., 2013; Yu et al., 2022). Considering that area acuity is consistently higher than number acuity during childhood (Odic et al., 2013), this should not affect the interpretation of the results in our study. However, it is important to acknowledge that future research should consider or control for other visual cues and education factors.

In conclusion, the study revealed a significant improvement in ANS acuity from grade one to grade three. However, no apparent differences were observed from grade three onwards. The presence of visual cues was found to enhance ANS acuity in older school-aged children when numerical cues were weakened, indicating the existence of distinct magnitude representational systems for numerical and non-numerical dimensions during development.

## Notes

1. The IES is calculated by dividing the correct response time by the accuracy rate, providing a measure of how efficiently individuals perform the task. It takes into account both speed and accuracy, allowing for a comprehensive evaluation of performance. A lower IES indicates greater efficiency, indicating that participants achieve higher accuracy within a shorter response time. In contrast, a higher IES suggests lower efficiency, reflecting slower response times or lower accuracy rates.

2. The numerical ratio between the two dot arrays determines the task difficulty, with a small ratio indicating a higher level of difficulty. It is noted that only adults from educated numerate cultures attain an average acuity with a ratio of 1.1 (Halberda & Feigenson, 2008). Therefore, trials with weak numerical cues (i.e., the ratio between 1.1 and 1.2) are considered more difficult compared to trials with strong numerical cues.
